# Continuous-flow catalytic asymmetric hydrogenations: Reaction optimization using FTIR inline analysis

**DOI:** 10.3762/bjoc.8.32

**Published:** 2012-02-23

**Authors:** Magnus Rueping, Teerawut Bootwicha, Erli Sugiono

**Affiliations:** 1 Institute of Organic Chemistry, RWTH Aachen University, Landoltweg 1, D-52074 Aachen, Germany

**Keywords:** asymmetric reduction, binolphosphoric acid, Brønsted acid, Hantzsch dihydropyridine, IR spectroscopy, real-time analysis

## Abstract

The asymmetric organocatalytic hydrogenation of benzoxazines, quinolines, quinoxalines and 3*H*-indoles in continuous-flow microreactors has been developed. Reaction monitoring was achieved by using an inline ReactIR flow cell, which allows fast and convenient optimization of reaction parameters. The reductions proceeded well, and the desired products were isolated in high yields and with excellent enantioselectivities.

## Introduction

In recent years, a growing interest in microreactor technology has been seen in the scientific community and the development of microfabricated reaction systems is actively pursued. Microreactor technology offers numerous advantages, including precise control of reaction variables, enhanced mixing quality, improved operational safety, reduced reagent consumption and ready scale-up of chemical processes. Due to the high surface-area-to-volume ratios of microstructured reactors, a high thermal rate and high portability of substrates can be achieved, which leads to improved product formation [1–42]. Furthermore, by incorporating inline analytical devices the progress of reactions can be monitored and analyzed in real time, allowing fast reaction screening and optimization [43–55].

Continuous flow microreactors have been applied to a number of standard transformations in organic synthesis [56–80]; however, examples regarding asymmetric reactions as well as organocatalytic reactions are scarce [81–96]. Herein, we present the first example of a continuous-flow organocatalytic asymmetric transfer hydrogenation performed in a microreactor. In this work a ReactIR flow cell was coupled with the microreactor and applied as an inline monitoring device for optimizing the reactions.

## Results and Discussion

**Scheme 1:**
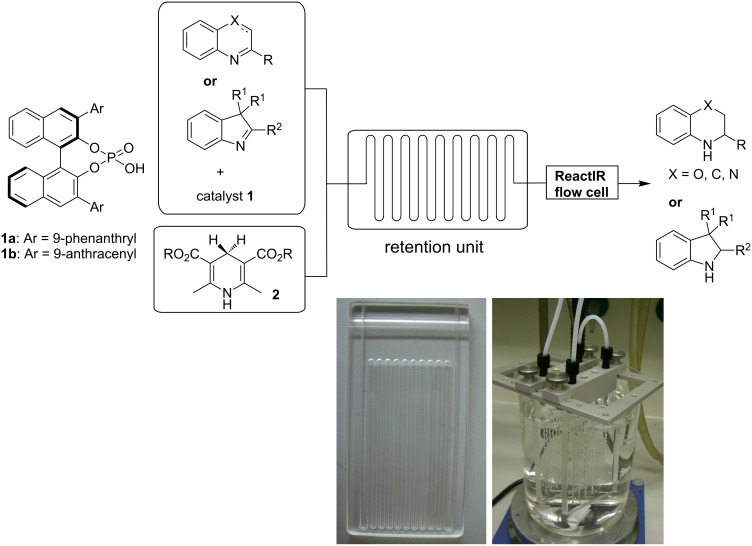
Experimental setup for the asymmetric transfer hydrogenation.

The continuous-flow microreactor system for the experiment was set up according to Scheme 1. The flow device was set up either with a single reactor, or with multiple reactors when a prolonged residence time was needed. The reagents were introduced separately, by using a syringe pump, through two inlets connected to Y-shaped connectors. The internal reaction temperature was monitored with an internal thermal sensor. The ReactIR 45m microflow cell equipped with a DiComp ATR (diamond-composite attenuated total reflection) probe was attached to the microreactor at the end of the reaction stream and was used as an inline analytical tool to determine the optimum reaction conditions. The IR spectra were recorded at predefined intervals and the raw data were analysed with iC-IR analysis software.

**Scheme 2:**
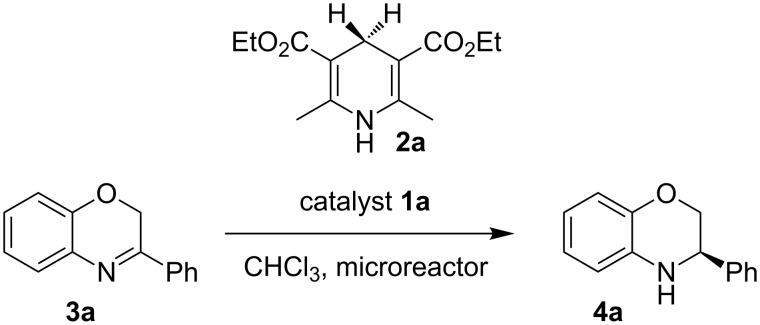
Asymmetric hydrogenation of benzoxazines.

The first reaction examined the asymmetric organocatalytic transfer hydrogenation [97–101] of benzoxazine **3a** in the presence of Hantzsch dihydropyridine **2a** as hydrogen source and a catalytic amount of chiral Brønsted acid **1a** (Scheme 2) [102].

**Figure 1:**
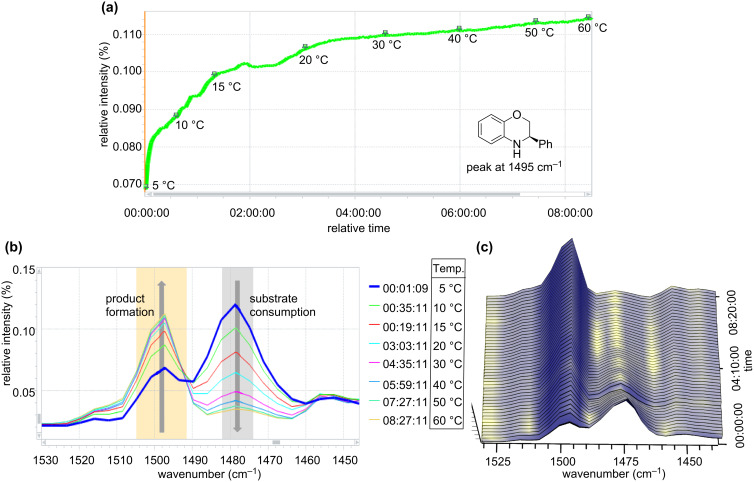
In situ ReactIR monitoring: (a) Trend curve of product formation at different temperatures. (b) Reaction spectra showing the consumption of the substrate and the formation of product at different temperatures. (c) Three-dimensional time-resolved spectral data.

Initial experiments were carried out at 0.1 mL min^−1^ flow rate in a commercial glass microreactor, which was attached to the ReactIR flow cell for in situ reaction monitoring. In order to control the reaction and to determine the use of educts and formation of product, reference spectra of the starting materials, solvents and reagents were recorded. Figure 1b and Figure 1c show real time IR spectra of the reaction mixtures after the subtraction of solvent in the spectral region of 1440 and 1530 cm^−1^. For direct inline analysis the signals at 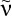 = 1479 cm^−1^ and 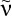 = 1495 cm^−1^ were ideal as they could easily be assigned to benzoxazine **3a** and dihydrobenzoxazine **4a**. Thus, in continuous flow the substrate consumption and product formation could readily be determined.

In order to find the optimal temperature for the asymmetric continuous-flow reduction, a temperature profile was recorded. The reaction temperature was initially 5 °C and was increased to 60 °C over a period of 8 h, while the conversion was monitored by inline IR-spectroscopy. Figure 1a shows the real-time plot of the peak intensity versus reaction time for the 1495 cm^−1^ absorption band at different temperatures. The trend-curve analysis by peak-height integration of this absorption band shows increased product formation with increasing temperature. By monitoring the signal change in this spectral region over the time of the reaction, the product formation (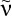 = 1495 cm^−1^) and substrate consumption (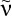 = 1479 cm^−1^) can be determined in real time. Analysis of the spectra provided us with an optimal temperature of 60 °C for this reaction. In general the IR-flow-cell technology is a good tool for in situ monitoring and provides a fast read out of reaction progress as the intensity of substrate and product peaks can be directly related to the conversion. Thus, as exemplified above, applying the inline analysis to different reaction parameters provides a fast and convenient method for reaction optimization.

By using the optimized reaction temperature and flow rate of 0.1 mL min^−1^, further experiments were conducted to examine the influence of the residence time on the conversion (Table 1). By performing the reaction with a residence time of 20 min, the product was isolated in 50% yield. With residence times of 40 min and 60 min, the product was isolated in 87% and 98% yields, respectively (Table 1).

**Table 1:** Optimization of the Brønsted acid catalyzed reduction of benzoxazines.^a^

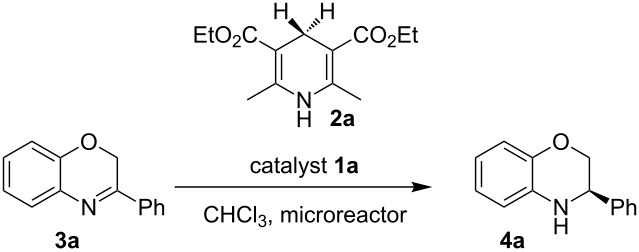				

Entry	**1a** [mol %]	Residence time [min]	Flow rate [mL min^−1^]	Yield [%]^b^

1	2	20	0.1	50%
2	2	40	0.1	87%
3	2	60	0.1	98%

^a^Reaction conditions: **3a**, **2a** (1.2 equiv), **1a** in CHCl_3_ (0.05 M) at 60 °C. ^b^Isolated yields after column chromatography.

Having found the optimum reaction conditions, we next investigated the scope of the Brønsted acid catalyzed reduction of 3-aryl-substituted benzoxazines **3** (Table 2). In general, 3-aryl benzoxazines **3** bearing either electron-withdrawing or electron-donating groups can be reduced in a continuous fashion and the products **4** were isolated in good yields and with excellent enantioselectivities.

**Table 2:** Scope of the Brønsted acid catalyzed reduction of benzoxazines.^a^

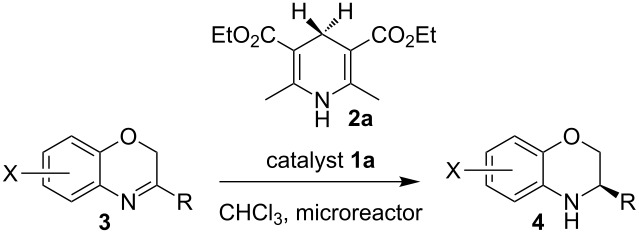			

Entry	Product **4**	Yield [%]^b^	ee [%]^c^

1	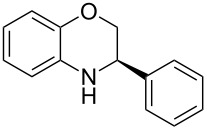 **4a**	98	98
2	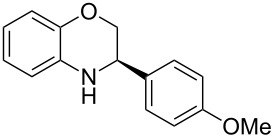 **4b**	96	97
3	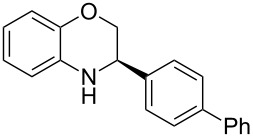 **4c**	98	98
4	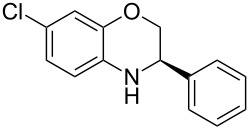 **4d**	81	97
5	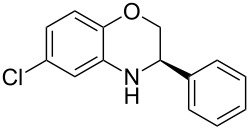 **4e**	85	99

^a^Reaction conditions: **3**, **2a** (1.2 equiv), 2 mol % **1a** in CHCl_3_ (0.05 M) at 60 °C, flow rate 0.1 mL min^−1^, residence time = 60 min. ^b^Isolated yields after column chromatography. ^c^Determined by chiral HPLC analysis.

Encouraged by the results, we next studied the transfer hydrogenation of quinolines **5** [103–106]. The optimum reaction temperature was determined according to the experiment described above. The effects of catalyst loading and residence time on the conversion and the enantioselectivity are summarized in Table 3. Performing the reaction at 60 °C with 5 mol % of Brønsted acid **1a** and residence time of 20 min afforded the desired product in 88% yield and 94% enantioselectivity (Table 3, entry 1). When the catalyst loading was reduced from 5 mol % to 2 mol %, a residence time of 40 min was found to be optimal to achieve comparable results (Table 3, entry 1 versus entry 2). A slight improvement of the conversion was observed by increasing the residence time to 60 min (Table 3, entry 3 versus entry 2). The catalyst loading can be decreased to 0.5 mol % without loss of reactivity and selectivity; the desired tetrahydroquinoline was isolated in 96% yield with 94% enantiomeric excess (Table 3, entry 5). A further decrease of catalyst loading to 0.1 mol % resulted in a significant drop in chemical yield, affording the product in lower yield while enantioselectivity was maintained (Table 3, entry 6).

**Table 3:** Optimization of the Brønsted acid catalyzed transfer hydrogenation of quinolines.^a^

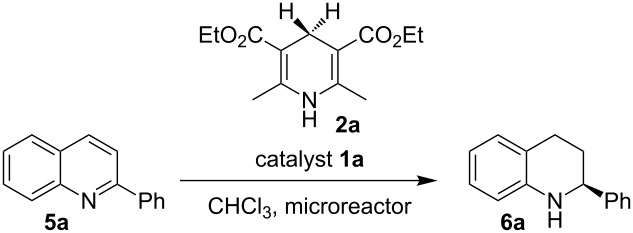					

Entry	**1a** [mol %]	*t* [min]	Flow rate [mL min^−1^]	Yield [%]^b^	ee [%]^c^

1	5	20	0.1	88	94
2	2	40	0.1	91	92
3	2	60	0.1	97	92
4	1	60	0.1	97	92
5	0.5	60	0.1	96	94
6	0.1	60	0.1	72	94
7^d^	0.5	60	batch	67	94

^a^Reaction conditions: **5a**, **2a** (2.4 equiv), **1a** in CHCl_3_ (0,1 M) at 60 °C, flow rate 0.1 mL min^−1^. ^b^Isolated yields after column chromatography. ^c^Determined by chiral HPLC analysis. ^d^Performed under batch conditions.

Although continuous-flow reactions provide many advantages, in certain cases it can be beneficial to conduct reactions under classical batch conditions. Therefore, we decided to carry out a direct comparison. Transferring the reaction conditions from continuous-flow to the batch showed a noticeable drop in conversion and the product was isolated only in 67% yield (Table 3, entry 5 vs entry 7). This observation is general, and typically lower reactivities were obtained. This can be explained by the better heat transfer in the microreactors as compared to the glass flask typically used in our batch reactions.

The scope and applicability of the method was then tested on various 2-substituted quinolines (Table 4). In general the asymmetric continuous-flow transfer hydrogenation of 2-substituted quinolines **5** proceeded well and afforded tetrahydroquinolines **6a**–**e** with excellent yields and enantioselectivities (Table 4).

**Table 4:** Scope of the Brønsted acid catalyzed transfer hydrogenation of quinolines.^a^

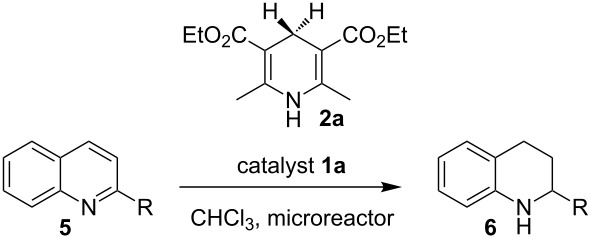			

Entry	Product **6**	Yield [%]^b^	ee [%]^c^

1	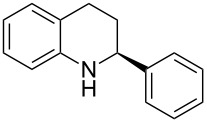 **6a**	96	94
2	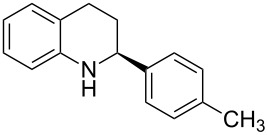 **6b**	91	96
3	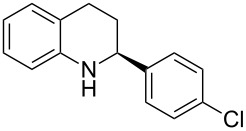 **6c**	94	99
4	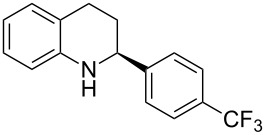 **6d**	91	99
5	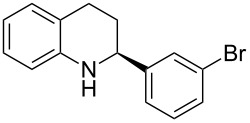 **6e**	97	96

^a^Reaction conditions: **5**, **2a** (2.4 equiv), 5 mol % **1a** in CHCl_3_ (0.1 M) at 60 °C, flow rate 0.1 mL min^−1^, residence time = 60 min. ^b^Isolated yields after column chromatography. ^c^Determined by chiral HPLC analysis.

Having established a protocol for a general and highly enantioselective transfer hydrogenation of quinolines, we decided to extend its scope to the reduction of quinoxalines **7** (Table 5) [107]. The asymmetric reduction of quinoxalines is typically more difficult to achieve. Using the optimized conditions for the fast inline reaction, we found that the continuous-flow reduction could be performed using 10 mol % Brønsted acid **1b**, a flow rate of 0.1 mL min^−1^ and 60 min residence time (Table 5).

**Table 5:** Scope of the Brønsted acid catalyzed transfer hydrogenation of quinoxalines.^a^

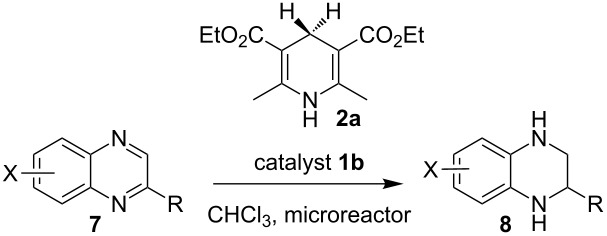			

Entry	Product **8**	Yield [%]^b^	ee [%]^c^

1	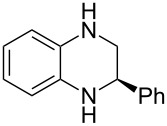 **8a**	77	90
2	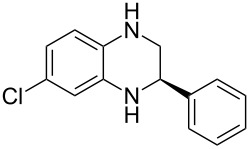 **8b**	68	84
3	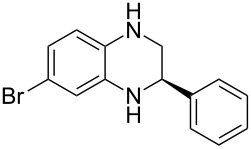 **8c**	53	86
4	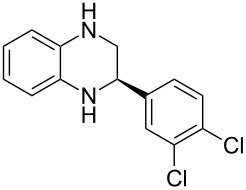 **8d**	86	94
5	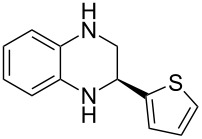 **8e**	41	76

^a^Reaction conditions: **7**, **2a** (2.4 equiv), 10 mol % **1b** in CHCl_3_ (0.1 M) at 60 °C, flow rate 0.1 mL min^−1^, residence time = 60 min. ^b^Isolated yields after column chromatography. ^c^Determined by chiral HPLC analysis.

To broaden the scope of the asymmetric hydrogenations in continuous flow further, the reduction of 3*H*-indoles **9** was studied (Table 6) [108]. Here the best reaction conditions turned out to be a temperature of 30 °C, a flow rate of 0.1 mL min^−1^, and a residence time of 20 min. The desired indolines **10** were isolated in good to high yields and with excellent enantioselectivities.

**Table 6:** Scope of the Brønsted acid catalyzed transfer hydrogenation of 3*H*-indoles.^a^

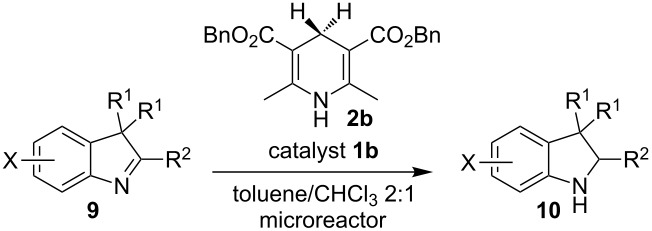			

Entry	Product **10**	Yield [%]^b^	ee [%]^c^

1	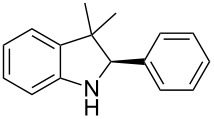 **10a**	95^d^	90
2	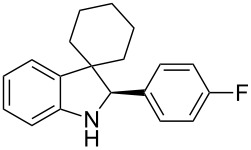 **10b**	88^d^ 98	98 98
3	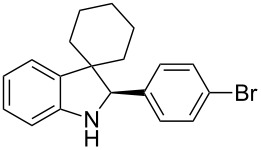 **10c**	60^d^ 96	99 99
4	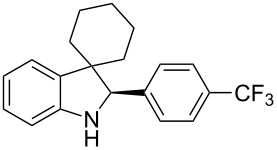 **10d**	78^d^ 95	99 99
5	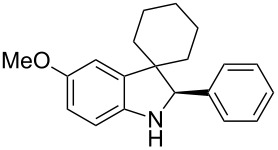 **10e**	94	97

^a^Reaction conditions: **9**, **2b** (1.3 equiv), 5 mol % **1b** in toluene/CHCl_3_ (2:1) (0.1 M) at 30 °C, flow rate 0.1 mL min^−1^, residence time = 20 min. ^b^Isolated yields after column chromatography. ^c^Determined by chiral HPLC analysis. ^d^Retention time: 10 min.

## Conclusion

In conclusion, we have demonstrated the potential of a microreactor setup coupled with FTIR inline analysis for monitoring asymmetric continuous-flow hydrogenations of benzoxazines, quinolines, quinoxalines and 3*H*-indoles. Following a real-time continuous-flow optimization, the corresponding products were obtained in good yields and with excellent enantioselectivities. By applying the FTIR inline monitoring, reaction parameters can be screened rapidly in a single reaction setup, and the optimal reaction conditions can be obtained much faster as compared to the classical sequence of conducting the reaction followed by analysis. Further work will include automated integration and feedback optimization of reaction parameters.
